# The SURFE^2^R (surface electrogenic event reader): from fundamental research to commercial product

**DOI:** 10.1007/s12551-025-01322-w

**Published:** 2025-06-16

**Authors:** Ronald J. Clarke

**Affiliations:** School of Chemistry, University of Sydney, Sydney, NSW 2006 Australia

**Keywords:** Capacitive coupling, Ion pumps, Membrane transporters, Solid-supported membrane, Electrophysiology, Rapid solution exchange

## Abstract

The purpose of this review is to describe the development of the Surface Electrogenic Event Reader (SURFE^2^R) instrument, from the discovery of its fundamental underlying principle of capacitive coupling of biological membranes in the late 1970s to the present-day commercial instrument, which since 2012 has been marketed by the company Nanion Technologies. The story of the SURFE^2^R’s development is a prime example of the transfer of a concept from fundamental research into a commercial product for the benefit of society. The capacitive coupling detection method was first recognized and used in research into the reaction mechanism of the proton pump bacteriorhodopsin from purple membrane fragments of a *Halobacterium*. The modern instrument now has a much wider application to research on the mechanisms of pumps and transporters in general, in the screening of drugs targeting pumps and transporters, and in quantifying drug affinity to biological membranes. The instrument is, therefore, of potential interest to researchers in both academia and the pharmaceutical industry. Because the author has worked and interacted with most, if not all, of the scientists involved in the evolution of the SURFE^2^R, the article also provides personal insights into the lives and careers of the leading scientists involved: Peter Läuger, Ernst Bamberg, Klaus Fendler, Thiemo Gropp and Niels Fertig.

## Introduction

**Fig. 1:**
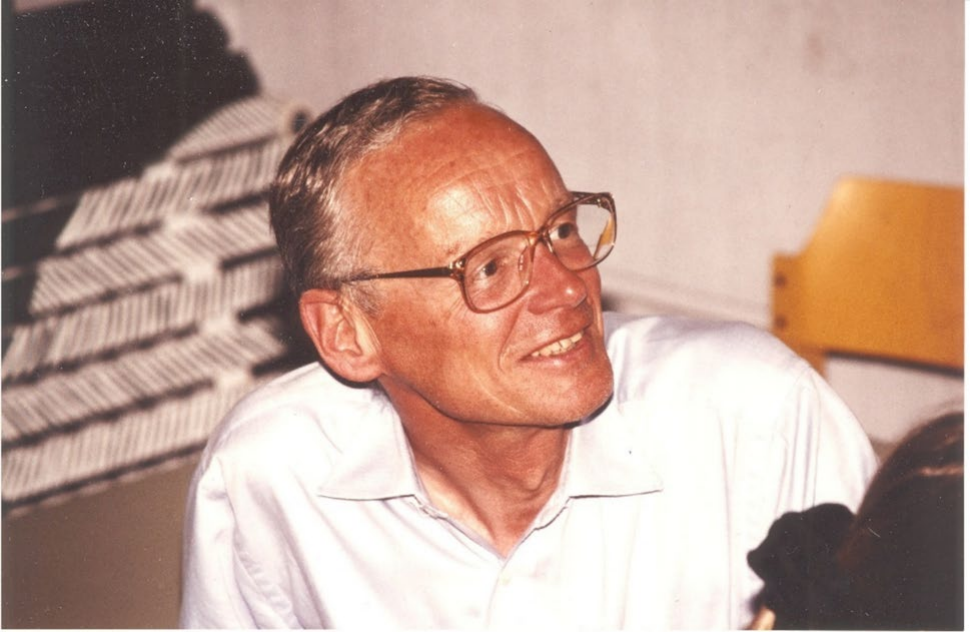
Peter Läuger (1934–1990) in the tea corner of the Department of Biophysics, Faculty of Biology, University of Constance, in 1989. Further details of his life and contributions to membrane biophysics can be found in Adam 1991 and Rigler and Henderson 1992

The Surface Electrogenic Event Reader (SURFE^2^R), which is produced and marketed by the company Nanion Technologies GmbH (Munich, Germany) is an instrument which has been specifically developed to measure the ion transport activity of ion pumps and membrane transporters, rather than that of ion channels. The instrument first came onto the market in 2006, at which time it was launched by the company IonGate Biosciences (Frankfurt am Main, Germany). The development of the instrument, however, goes back over 40 years earlier to the late 1970s, when the fundamental principle on which the instrument’s detection method is based, capacitive coupling via membrane adsorption, was first described in the context of membrane transport in a paper from the group of Professor Peter Läuger (University of Constance, Germany) (see Fig. 1) and collaborators from Basel and Jülich, in Switzerland and Germany, respectively (Bamberg et al 1979). The purpose of this review is to provide the story of the evolution of the SURFE^2^R instrument, as a prime example of how an idea, which originated in fundamental research has led after many years to a commercial product, from which researchers of ion pumps and transporters around the world can benefit. Although I have not been involved in the development of the instrument, I have followed all the stages of its evolution since 1987, when I came to Professor Läuger’s laboratory as a postdoc. Because I have been a colleague of almost all the key players in the steps leading to the SURFE^2^R as it exists today and know them both on a personal as well as a professional level, I have chosen to write this story of the instrument’s invention and transfer from academia to industry in the first person, firstly because it seems to me to be more appropriate due to the close connection I have had with the people involved but also to give the reader more insight into the human side of scientific research. In addition, because the development of the instrument occurred in Germany and I was embedded in German society during much of this time, I will refer to the scientists involved in the way that I knew or know them according to German custom and the German sense of courtesy.

## SURFE^2^R origins—Bacteriorhodopsin

**Fig. 2:**
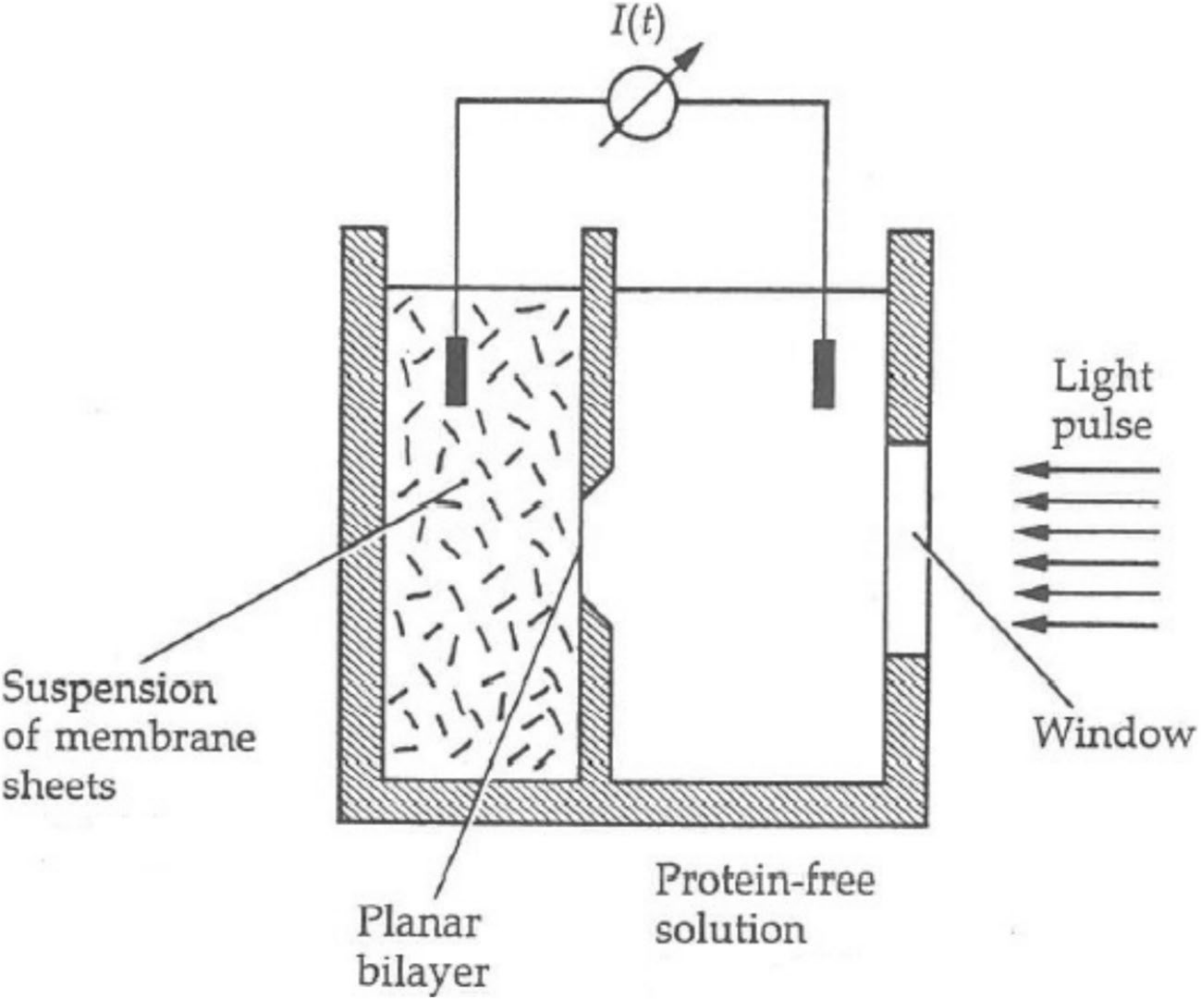
Set-up for the measurement of current signals from a bilayer lipid membrane with adsorbed membrane sheets (e.g., purple membranes with bacteriorhodopsin or Na^+^,K^+^-ATPase membrane fragments). The diameter of the circular membrane is about 1–2 mm. The solutions are connected to the external measuring circuit via silver/silver chloride electrodes. The volume of the compartment with membrane sheets is 0.3 ml. That of the protein-free solution compartment is 5.0 ml. A magnetic stirrer bar (not shown) is included in the compartment with membrane sheets. Reproduced with permission of Oxford Publishing Limited through PLSclear. from P Läuger (1991), Electrogenic Ion Pumps, Sinauer Associates, Sunderland, Massachusetts, USA, pp 114–115

**Fig. 3:**
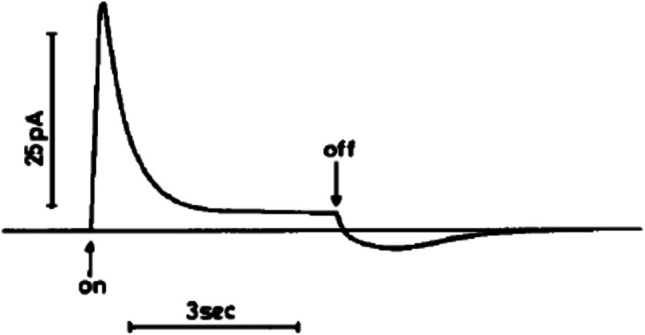
Time-course of the electrical photo-response of a bilayer lipid membrane with bacteriorhodopsin purple membrane fragments added. “On” and “off” refer to the switching on and off of white-light illumination. (Reproduced from Dancsházy and Karvaly 1976 with permission from John Wiley and Sons). Looking at the initial peak of the transient current, when the light is turned on, the peak of the transient current reaches a maximum of approximately 30 pA. This is on top of a constant stationary current of approximately 3 pA, to which the transient current decays after 2–3 s in this case. Thus, approximately 93% of the peak current is due to the capacitive component

The origins of the SURFE^2^R instrument go back to measurements on the light-driven proton pump bacteriorhodopsin. To measure the kinetics of charge transport across lipid membranes by bacteriorhodopsin, several groups (Dancsházy and Karvaly 1976; Drachev et al 1978; Herrmann and Rayfield 1978; Bamberg et al 1979; Seta et al 1980; Fahr et al 1981) carried out experiments in which they added either lipid vesicles incorporating purple membrane patches from *Halobacteria* or purple membranes alone to a solution bathing a bilayer lipid membrane (BLM). The experimental set-up is shown in Fig. 2. On illumination with either white light, a flash lamp or laser with a wavelength which is absorbed by the retinal chromophore of bacteriorhodopsin, they observed transient currents which could be attributed to the proton pumping activity of bacteriorhodopsin. An example is shown in Fig. 3.

**Fig. 4:**
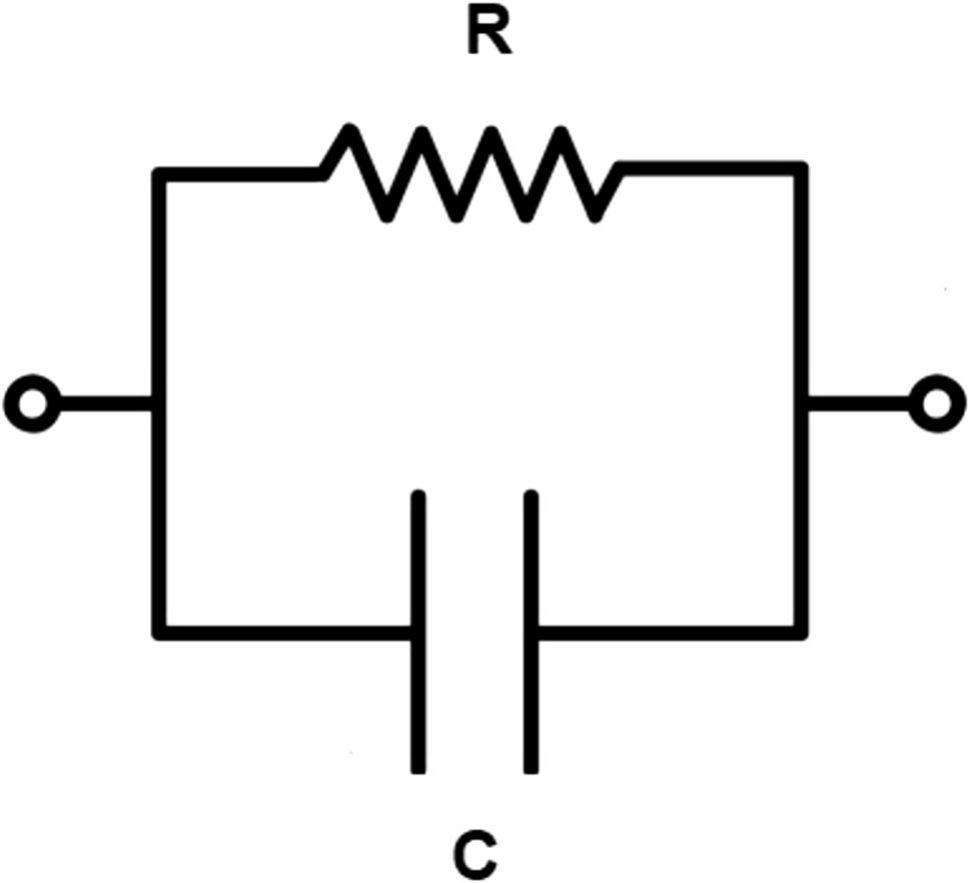
Equivalent circuit diagram of a lipid membrane. The membrane has simultaneously the properties of electrical resistance and capacitance. It can be described as a resistor, R, and capacitor, C, in parallel

To understand the origin of currents such as those shown in Fig. 3 requires first an appreciation of the electrical properties of a lipid membrane. The presence of a lipid membrane between two electrolyte solutions creates a barrier to the movement of ions from one solution to the other, but the barrier is not completely impermeable to ions. The membrane, therefore, behaves in part as a resistor. However, equal and opposite charges can also build up on each surface of the membrane, i.e., acting as a capacitor. Thus, the membrane simultaneously possesses the properties of both a resistor and a capacitor. A lipid membrane can, therefore, be described by an equivalent circuit diagram consisting of a resistance and capacitance in parallel (Adam et al. 1988; Hille 2001) (see Fig. 4). The current which passes through the membrane, i.e., through the resistance component of the membrane, has a constant value and is, therefore, termed a *stationary current*. The capacitance component of the membrane also allows current to flow, but not via charges moving through the membrane, instead by ions binding to one surface of the membrane and ions of the same charge moving away from the opposite surface of the membrane. Thus, as a positive charge builds up on one surface of the membrane, a negative charge builds up on the opposite surface. A current is observed only as long as charge is binding to the membrane surface. When the membrane is saturated with ions, or, in other words, the membrane capacitor is fully charged, the current ceases. The current through the capacitance component of the membrane is, therefore, not constant. Initially, when the membrane is connected to a power source and no ions have bound to the membrane, the current rises quickly from zero to a maximum. Then, as the charge builds up on the membrane, the current gradually decays to zero. *Capacitive currents* are, therefore, biphasic transient currents, with a rapid rising phase followed by a slower decay phase. This contrasts with the constant stationary current through the resistance component of the membrane. Further theoretical details relevant to the functioning of the SURFE^2^R instrument are described at the end of the paper.

**Fig. 5:**
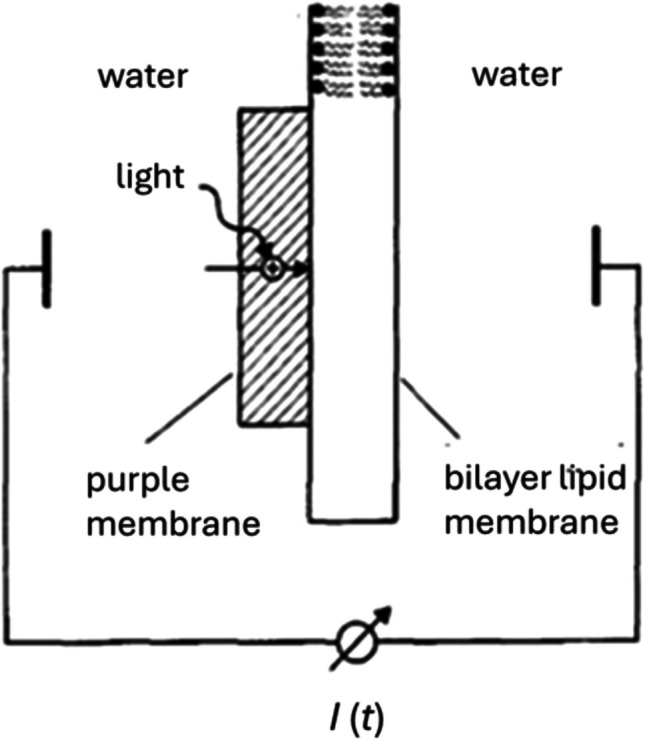
Compound membrane system consisting of a bilayer lipid membrane with adsorbed purple membrane sheets of bacteriorhodopsin. Activation by a light flash causes a transient ion pump current *I*_*p*_(*t*), which is exactly compensated for by a time-dependent current *I*(*t*) in the external measuring circuit. (Modified from Bamberg et al 1979)

Initially, researchers applying the method shown in Fig. 2 attributed the observed transient currents to bacteriorhodopsin molecules incorporated into the BLM (Dancsházy and Karvaly 1976; Drachev et al 1978). Herrmann and Rayfield (1978), using bacteriorhodopsin purple membrane fragments reconstituted into lipid vesicles, first proposed that a significant proportion of the transient current signal originated in a capacitive (or displacement) current, but they were still of the opinion that one side of the vesicles fused to the BLM. Bamberg et al (1979) also carried out experiments using bacteriorhodopsin purple membrane fragments but without reconstituting them into lipid vesicles. They found that, even in this system, transient current signals were produced on illumination of bacteriorhodopsin. If the membrane fragments had been incorporated into the membrane one would have expected the transmembrane proton pumping by bacteriorhodopsin to have increased the *stationary* current. This was not the case. Bamberg et al (1979) therefore concluded that the membrane fragments were adsorbing onto the BLM surface and producing transient signals via coupling of the BLM and purple membrane fragment capacitances. They also concluded that the purple membranes must be adsorbing to the BLM with a preferential orientation, i.e., one surface of the purple membrane prefers to interact with the BLM more frequently than the other. If the orientation of the purple membranes was random, one would expect currents in both directions so that there would be zero net current. Thus, a compound membrane system is formed with a sandwich-like structure consisting of a purple membrane resting on the BLM (see Fig. 5). Bamberg et al. (1979) also performed measurements after adding gramicidin A to the solution in the compartment on the opposite side of the BLM to where the purple membrane fragments of bacteriorhodopsin had been added. In the presence of gramicidin A, the current caused by bacteriorhodopsin illumination massively increased, but the major effect was an increase in the stationary (i.e., transmembrane) current, on top of which an initial transient current was still superimposed. Because gramicidin A is a channel-former which increases the BLM permeability to protons, they concluded that the stationary current was due to gramicidin A allowing protons which had been pumped by bacteriorhodopsin across the purple membrane to then pass further through the BLM.

The compound membrane shown in Fig. 5 acts as two capacitors in series, with one capacitance associated with the purple membrane and one with the underlying BLM. In an analogous electrical circuit with two capacitors and a voltage power supply, current will only flow as long as the capacitors are being charged. In the same way, the current produced in the external measuring circuit of the BLM set-up is transient and decays to zero when the membrane capacitors are fully charged. This is usually the case because the electrical resistances of the purple membrane and the BLM are normally extremely high, so that no stationary currents across the membranes are possible. Only when channel formers or ionophores are added will a stationary current be observed. In this experimental set-up the light source replaces the electrical power supply, because it is the light source which generates the initial charge movement in bacteriorhodopsin. Further discussion of the principle of the capacitive coupling technique is provided later, after the historical development of the method has been presented.

Now come the most important questions. What is the value of this experimental technique? What can we learn from such measurements? The measured current in the external circuit exactly compensates for the current arising from charge movement within the bacteriorhodopsin molecule, and the charge movement within the bacteriorhodopsin molecule is due to an electrogenic reaction step of the protein’s proton-pumping photocycle. This could be due to a light-induced conformational change of the bacteriorhodopsin molecule which causes a bound proton to move a certain distance across the membrane, or it could originate from a conformational change where charged amino acid residues of the protein move relative to the membrane surface. Therefore, the measurements provide detailed kinetic information on the mechanism of bacteriorhodopsin’s ion pumping mechanism, if the reaction step of the bacteriorhodopsin pump cycle responsible for the observed transient can be identified. This, of course, is fundamental scientific information and the practical value may not seem immediately obvious, but the applications of the capacitive coupling technique will become clear in later sections. For now, we will concentrate on the development of the technique.

### Steps on the way to the SURFE^2^R—Ion pump activation

Before proceeding further to discuss the application of capacitive coupling to other membrane transport systems and the further evolution of the SURFE^2^R instrument, it is important here to make clear the different types of transport proteins. Bacteriorhodopsin is classified as a *primary* active transport system. What this means is that it pumps ions against an electrochemical potential gradient, i.e., it requires energy, and it derives this energy directly from light, in Nature from the sun. Other examples of active transport systems are ion-motive ATPases, such as the Na^+^,K^+^-ATPase, which derives its energy from the hydrolysis of ATP to ADP and inorganic phosphate. In contrast, *secondary* active transporters utilize the energy stored in an electrochemical potential gradient that has already been created by a primary active transport system. An example is the Na^+^/glucose co-transporter, which utilizes the energy of the Na^+^ electrochemical potential gradient created across the plasma membrane of a cell by the Na^+^,K^+^-ATPase to absorb glucose into the cytoplasm. Thus, bacteriorhodopsin can be activated by light, the Na^+^,K^+^-ATPase requires ATP for its activation, and the Na^+^/glucose co-transporter requires Na^+^ ions to create a glucose concentration gradient across the membrane.

**Fig. 6:**
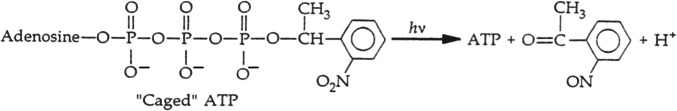
Photochemical release of ATP from P3-1-(2-nitrophenyl)ethyl ATP (NPE-caged ATP), developed by Kaplan et al (1978)

The capacitively-coupled BLM set-up shown in Figs. 2 and 5 is excellent for the activation of a light-activated ion pump like bacteriorhodopsin, but to activate an ATP-dependent system such as the Na^+^,K^+^-ATPase one requires a rapid addition of ATP. It is not possible to simply add ATP to one cell compartment with a pipette. Firstly, this would be too slow to enable any fast kinetic measurements and secondly, the BLM is very fragile and could easily rupture when solution additions or exchanges are made. Therefore, instead, researchers turned to the use of photochemically active ATP derivatives which release ATP after photolysis with a laser flash, so-called caged ATPs (see Fig. 6) (Kaplan et al 1978; McCray et al 1980).

**Fig. 7:**
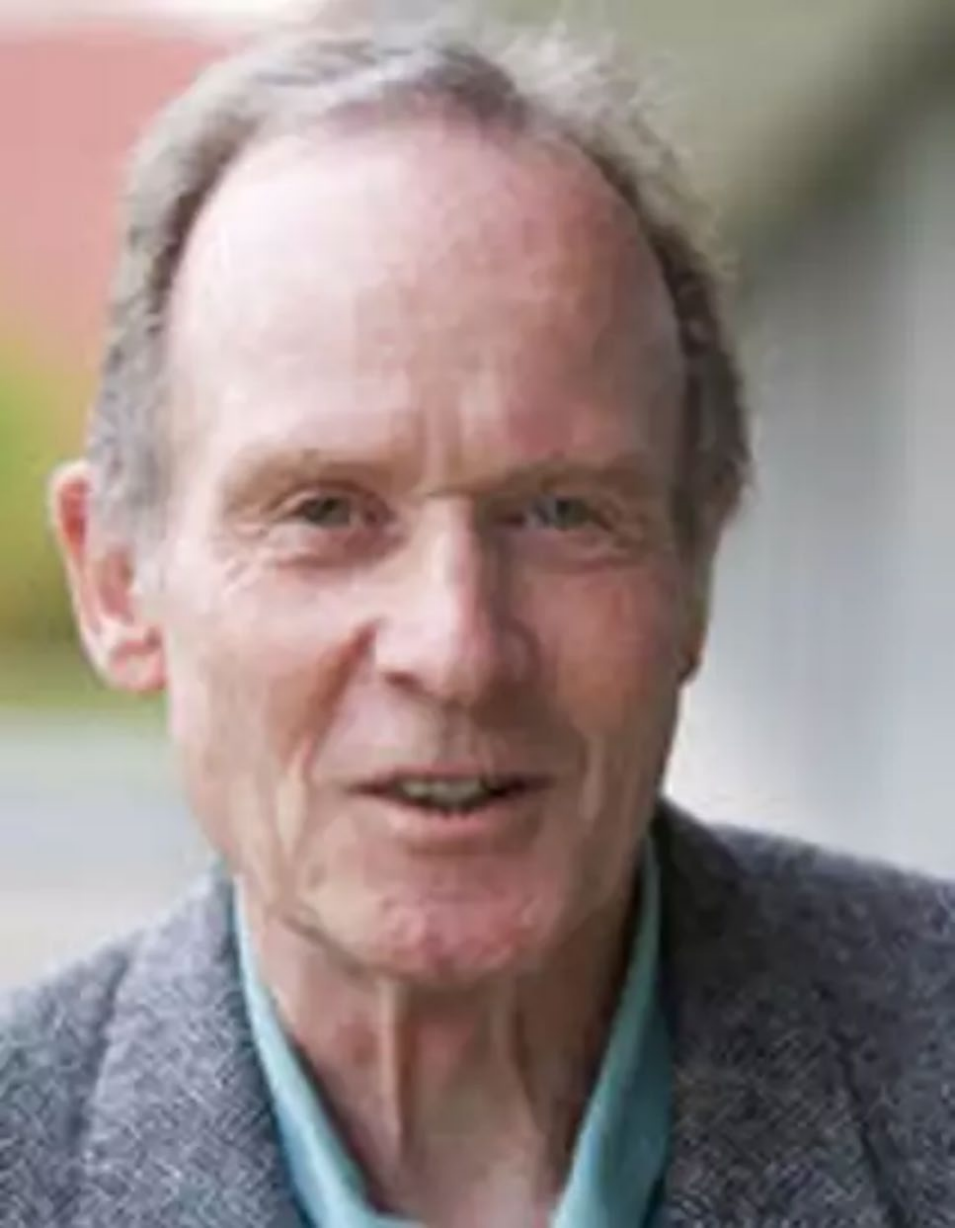
Ernst Bamberg, Department of Biophysical Chemistry, Max-Planck-Institute of Biophysics, Frankfurt/Main, Germany (reproduced with permission from the Max-Planck-Institute of Biophysics, Frankfurt/Main, Germany)

The next major player along the road to the development of the SURFE^2^R instrument was Ernst Bamberg (see Fig. 7), who applied caged ATP in conjunction with capacitive coupling. His name has already been mentioned in the previous section due to his first authorship on the paper describing the application of capacitive coupling to bacteriorhodopsin (Bamberg et al 1979). Although *Herr* Läuger and *Herr* Bamberg were both born in Germany, both spent many years of their lives criss-crossing the border between Germany and Switzerland. Both completed their PhDs in physical chemistry at the University of Basel in Switzerland, adjacent to the very south-western border of Germany’s state of Baden-Württemberg. The University of Basel had then, as now, a strong reputation for research and teaching in biophysical chemistry. *Herr* Bamberg graduated with a PhD there in 1971 but was already working with *Herr* Läuger at the newly established University of Constance in 1969. *Herr* Läuger was one of the early appointments there as a Professor of Biology in its Department of Biophysics, Faculty of Biology, in 1968. The University of Constance had only been founded in 1966 and a new campus on a hill overlooking Lake Constance was opened in 1972. Like Basel, Constance is directly on the German-Swiss border, and *Herr* Läuger in fact lived in Kreuzlingen, on the Swiss side of the lake, crossing the border every day to come to work at the University.

From its beginnings the University of Constance was designed as a research-intensive university. *Herr* Läuger and *Herr* Bamberg worked closely together there for over 10 years, with *Herr* Bamberg completing his *Habilitation*, the second German doctorate, required to qualify as a university professor, in 1976, and being awarded a Heisenberg fellowship by the German Research Council (*Deutsche Forschungsgemeinschaft*) in 1979. This is a fellowship for researchers which recognizes their suitability for an academic career and appointment to a German professorship. Together, *Herr* Bamberg and *Herr* Läuger had a fruitful working relationship, publishing 16 joint papers between 1969 and 1981, with to-date over 1,600 citations. The majority of these were on passive and active transport across bilayer lipid membranes, by gramicidin and bacteriorhodopsin, respectively. This productive collaboration was probably aided by the fact that they were very different characters. *Herr* Läuger had a great awareness of experimental techniques and how they could be applied, but his greatest talent lay in his grasp of physicochemical theory and its application to the mathematical explanation of experimental results. Without doubt he belonged to the best theoretical biophysicists of his generation. Ironically, although I never saw *Herr* Läuger perform an actual experiment, when he was in the Biophysics Department at the University, he would always be dressed in an immaculately white lab coat. The solitude and deep concentration needed to develop new theories and correctly apply existing ones probably suited his character well, which one could describe as shy and modest, but at the same time kind and generous, coupled with nervous energy, which he could channel into his scientific productivity as well as his hobbies. *Herr* Bamberg’s talents, on the other hand, lay more in the design and performance of experiments, later expanding the scope of his research into new fields such as cell biology and molecular genetics, and even creating a new field, i.e., optogenetics. He believed that, just because he worked in a biophysics department or later in a biophysics institute, this didn’t mean that he should restrict his research activities purely to biophysical experiments. What was important in his view was to solve a problem, and if that meant venturing into other fields, so be it. He also had a more outgoing personality than *Herr* Läuger, although *Herr* Läuger was very friendly, very knowledgeable and a good discussion partner once you got to know him.

**Fig. 8:**
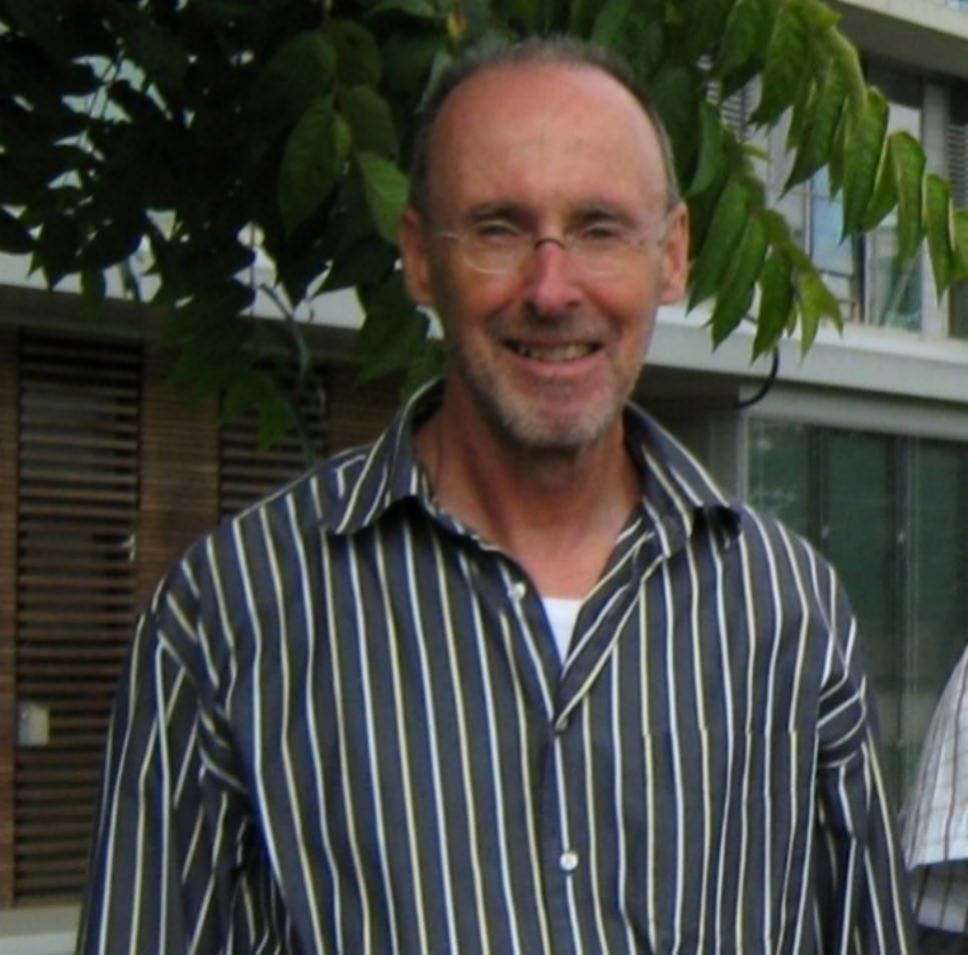
Klaus Fendler, in September 2014, standing in front of the new Max-Planck-Institute of Biophysics, on the Riedberg campus of the Johann-Wolfgang-von-Goethe-University of Frankfurt, Germany (photo provided by Andre Bazzone)

In 1983 *Herr* Bamberg was appointed as an independent group leader at the Max-Planck-Institute of Biophysics in Frankfurt/Main, being promoted 10 years later, in 1993, to Director of the Department of Biophysical Chemistry. When he moved to Frankfurt, *Herr* Bamberg took with him a PhD student, Georg Nagel, who had been studying biology and biophysics in Constance. But *Herr* Bamberg needed more people to build up his group. Georg recommended a physicist friend of his, Klaus Fendler, who had also been studying in Constance. So, Klaus and Georg ended up moving together with *Herr* Bamberg to Frankfurt. Klaus (see Fig. 8) was to play the most decisive role in the development of the SURFE^2^R instrument and can rightly be regarded as its inventor.

**Fig. 9:**
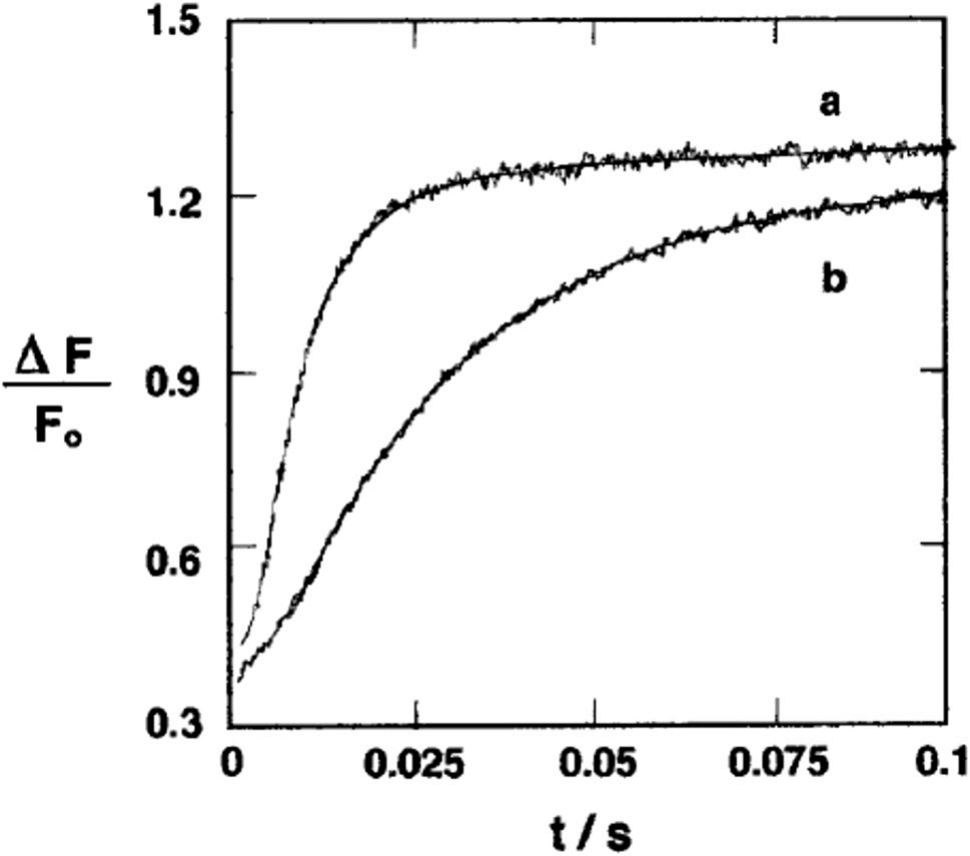
Stopped-flow fluorescence transients of native Na^+^,K^+^-ATPase membrane fragments from rabbit kidney noncovalently labelled with RH421 (75 nM, after mixing). Na^+^,K^+^-ATPase (10 μg/ml or 0.068 mM, after mixing) was rapidly mixed with an equal volume of Na_2_ATP (25 μM, after mixing). Each solution was in a buffer containing 130 mM NaCl, 30 mM imidazole, 5 mM MgCl_2_, and 1 mM EDTA; pH 7.4, *T* = 24 °C. The fluorescence of membrane-bound RH421 was measured using an excitation wavelength of 577 nm at emission wavelengths ≥ 665 nm (RG665 glass cutoff filter). The solid lines represent fits to a sum (either one or two) of exponential time functions. (*a*) In the absence of NPE-caged ATP. The calculated reciprocal relaxation times were 137 (± 3) s^−1^ (93% of the total amplitude) and 17 (± 4) s^−1^ (7%). (*b*) Before mixing with ATP, the enzyme was equilibrated with NPE-caged ATP (125 μM, after mixing). The calculated reciprocal relaxation time was 37 (± 1) s^−1^. Reprinted from the Biophysical Journal, 75, R. J. Clarke, D. J. Kane, H.-J. Apell, M. Roudna and E. Bamberg, Kinetics of Na^+^-dependent conformational changes of rabbit kidney Na^+^/K^+^-ATPase, 1340–1353. Copyright 2025, with permission from Elsevier

In Frankfurt *Herr* Bamberg continued his work on retinal proteins, such as bacteriorhodopsin. In 2002, almost 20 years after the move to Frankfurt, Georg Nagel discovered the channelrhodopsins (Nagel et al 2002) and, with *Herr* Bamberg, laid the foundations for the new field of optogenetics, for which both are now most famous. However, for the development of the SURFE^2^R more importantly, *Herr* Bamberg also started work on the kinetics of the Na^+^,K^+^-ATPase or sodium pump. One of the important physiological roles of the Na^+^,K^+^-ATPase is in the kidney where it produces the Na^+^ electrochemical potential gradient necessary to drive the reabsorption of nutrients into the bloodstream. Therefore, the Na^+^,K^+^-ATPase is naturally very highly expressed in the kidney, and it is, therefore, possible to prepare membrane fragments rich in Na^+^,K^+^-ATPase, analogous to the purple membrane fragments of *Halobacteria*, with a high protein content (Jørgensen 1974). These Na^+^,K^+^-ATPase membrane fragments are ideal for capacitive coupling with a black lipid membrane. *Herr* Bamberg’s group, with the experiments led in this case by Klaus Fendler, published their first paper (Fendler et al 1985) on the kinetics of the partial reactions of the Na^+^,K^+^-ATPase in 1985. The experimental results of Fendler et al (1985) were later confirmed by *Herr* Läuger’s group in Constance (Borlinghaus et al 1987; Apell et al 1987). The Frankfurt group found (Nagel et al 1987), however, that the magnitudes of the transient currents depended not only on the ATP concentration, but also on the ATP/caged ATP ratio. They attributed this to the binding of unphotolysed caged ATP to the ATP binding site of the Na^+^,K^+^-ATPase, hence blocking the site for ATP and causing inhibition of the signal. This was confirmed just over 10 years later (Clarke et al 1998) using an independent rapid reaction technique, namely stopped-flow fluorimetry, using the voltage-sensitive fluorescent dye RH421 to detect partial reactions of the Na^+^,K^+^-ATPase (see Fig. 9).

The use of caged ATP in capacitively-coupled transient current measurements is, therefore, a serious concern. The problem lies in the fact that, after a laser flash, not all the caged ATP is photolysed into ATP. If the inhibition of the Na^+^,K^+^-ATPase by unphotolysed caged ATP is not taken into consideration in the data analysis, then the observed rate constants that one derives will be artificially low. Therefore, one has two options. One is to correct for caged ATP inhibition. The other is to avoid using caged ATP altogether. In the SURFE^2^R instrument no caged compounds are used at all.

### Going with the flow

**Fig. 10:**
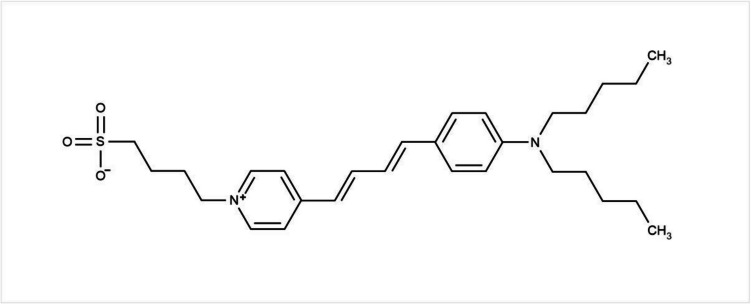
Structure of the voltage-sensitive styrylpyridinium dye RH421. The localized negative charge on the sulphonate group acts as a hydrophilic anchor, fixing this part of the molecule at the membrane/aqueous solution interface. The two alkyl chains at the other end of the molecule insert themselves into the hydrocarbon interior of the membrane, and the molecule’s aromatic fluorophore is located within the lipid headgroup region of the membrane. The fluorophore’s positive charge is delocalized between the pyridinium and amino nitrogens. In the ground state the positive charge lies more on the pyridinium nitrogen, as shown in the figure. On electronic excitation, there is a large charge shift, with the positive charge moving towards the amino nitrogen. This makes the dye’s UV/visible absorption spectrum and its fluorescence excitation spectrum very sensitive to the local electric field (Loew et al 1978). Reprinted from Baumgart A, Le DT, Cranfield CG, Bridge S, Zerlotti R, Palchetti I, Tadini-Buoninsegni, Clarke RJ (2025) Membrane binding of hydrophobic ions: Application of new kinetic methods. Langmuir 41:8081–8091. Copyright 2025 American Chemical Society

If one wishes to avoid using caged ATP for the activation of phosphorylation-dependent reactions of membrane transport ATPases, the only option is to add ATP directly via a rapid mixing device, such as a stopped-flow system. This is what was used to obtain the results shown in Fig. 9. However, because of the fragility of a BLM, rapid mixing and the BLM are incompatible. The solution adopted for the experiments shown in Fig. 9 was not to use a BLM at all. Instead, the Na^+^,K^+^-ATPase membrane fragments were noncovalently stained with a fluorescent voltage-sensitive dye, RH421 (see Fig. 10), which converts a charge movement associated with the ATPase mechanism into an observable fluorescence signal. Such dyes were introduced to the Na^+^,K^+^-ATPase field by Irena Klodos from the Department of Biophysics at the University of Aarhus, Denmark. This was the same department where Jens Christian Skou, the discoverer of the Na^+^,K^+^-ATPase and the winner of the 1997 Nobel Prize in Chemistry worked. Irena carried out her first experiments (Forbush and Klodos 1991) applying the RH dyes to the Na^+^,K^+^-ATPase while visiting the laboratory of Biff Forbush at the Yale School of Medicine in the USA. On a conference visit to Aarhus, *Herr* Läuger heard Irena talk about her still-unpublished results on the Na^+^,K^+^-ATPase using the RH dyes. He was quick to realise their potential and, on returning to Constance, introduced them to his own laboratory (Bühler et al 1991). In these studies, the Constance group, however, still activated the Na^+^,K^+^-ATPase via photochemical release of ATP from caged ATP. So, the problem of inhibition by unphotolysed caged ATP was still an issue. Not only that, it was later found from stopped-flow measurements (Kane et al 1997) in the Bamberg group in Frankfurt that RH421 at micromolar concentrations can also inhibit the Na^+^,K^+^-ATPase. In fact, this is to be expected, because the dye must have charges to respond to changes in membrane local electric field strength caused by charge movements associated with the protein, but the charges of the dye would also create their own electric fields, which would influence the kinetics of charge movements of the protein. To measure the correct values of rate constants of the protein’s partial reactions, the dye concentration must, therefore, be kept as low as possible.

Apart from measuring the values of rate constants, the determination of which partial reactions of the Na^+^,K^+^-ATPase are responsible for transient currents or for RH421 fluorescence changes can provide valuable information about structural changes involved in the ion pumping mechanism. So, for example, one could use RH421 simply as a probe that produces a fluorescence change to follow the kinetics of the protein, but if one could resolve the response mechanism of the dye, this could provide important insights into the pumping mechanism. In 2017, my own group presented evidence (Garcia et al 2017) that, rather than detecting electric fields from the transported ions, RH421 detects a Na^+^,K^+^-ATPase conformational change at the membrane surface. We attributed this to a movement of the polybasic N-terminus (positively charged) of the protein’s catalytic α-subunit on and off the negatively charged (due to negatively charged phosphatidylserine headgroups) cytoplasmic surface of the membrane. Not only might this be an as yet unrecognized feature of the enzyme’s mechanism, it could also be a site of regulation of its activity via an electrostatic switch mechanism involving phosphorylation of the N-terminus by a protein kinase (Clarke 2023a, b). This is already a well-established regulatory mechanism for peripheral membrane proteins (McLaughlin and Aderem 1995), but not yet for integral membrane proteins.

I will now make a brief diversion from ion pump kinetics into structure. Although this is not directly relevant to the SURFE^2^R, functional and structural studies in the membrane biophysics field now go hand in hand, so it is worthwhile considering parallel advances in structural biology that were happening over the same timeframe as the SURFE^2^R was being developed. When I was working in *Herr* Läuger’s laboratory in the late 1980s the idea of having a crystal structure of the Na^+^,K^+^-ATPase was in the air. Hartmut Michel, Johann Deisenhofer and Robert Huber had been awarded the Nobel Prize in Chemistry in 1988 for determining the structure of the bacterial photosynthetic reaction centre, the first high resolution structure of an integral membrane protein ever to be determined (Deisenhofer et al. 1985). If the structure of the Na^+^,K^+^-ATPase could only be determined, what secrets this would reveal about the mechanism of the protein. Unfortunately, *Herr* Läuger did not live to see this day. In August 1990, he travelled to Vancouver to attend the triennial congress of the International Union of Pure and Applied Biophysics (IUPAB). Being the German representative on the IUPAB Council, he also took part in the IUPAB Council Meeting held during the congress. At the congress he held a lecture which was very well received by the attendees. From Vancouver he travelled to Woods Hole, Massachusetts, for the 44th annual meeting of the Society of General Physiologists. Immediately after the Woods Hole meeting he flew to Venezuela to go hiking in the Andes, and, being a passionate amateur botanist, in particular to see and photograph the mountain flora there. Tragically, on the 13th of September near Mérida he was killed in a mountain climbing accident. *Herr* Läuger’s death was a massive shock and a huge loss to the biophysics community. For me personally, it was the loss of a good friend and mentor. He did, however, leave an invaluable legacy to the membrane biophysics field. In 1987 he was the Distinguished Lecturer for the Society of General Physiologists at the IUPAB Congress in Jerusalem, and he was asked at that time to write a monograph for the Society. In August 1990 he’d already submitted the first half of the manuscript for his book entitled *Electrogenic Ion Pumps*. At the Society’s meeting in 1990 he informed them that the remaining chapters were complete and would be submitted in a few weeks, when he returned to Constance. In the end, the final drafting and proofreading of the manuscript were completed by his longtime collaborator and former student Hans-Jürgen Apell. Hans-Jürgen also took over the task of supervising the Läuger membrane biophysics group in Constance and continued the direction of research on the Na^+^,K^+^-ATPase. *Electrogenic Ion Pumps* is superbly written and illustrated. To this day it serves as a standard work and essential reading for anyone working on ion pumps. The first high resolution structure of a P-type ATPase, the sarcoplasmic reticulum Ca^2+^-ATPase, didn’t appear until 2000 (Toyoshima et al 2000), and the first Na^+^,K^+^-ATPase structure came out in 2007 (Morth et al 2007). These structures, and others since, have been very helpful in understanding how ion pumps of the P-type class work, but, being static pictures, they don’t explain everything. Functional studies such as those performed using the SURFE^2^R instrument are still very valuable.

### Der Kindersarg: The SURFE^2^R prototype

By the early 1990s several disadvantages of the use of BLMs in conjunction with capacitive coupling were clear. Firstly, because of their fragility, BLMs are only stable when spread over a small surface area of approximately 0.01 cm^2^. If a membrane could be formed on a solid substrate, on the other hand, much larger surface areas could potentially be achieved, so that many more pumps or transporters could be measured simultaneously, leading to much greater currents and hence larger signal-to-noise ratios. This is a very important consideration for pumps and transporters because their turnover numbers are relatively low in comparison to ion channels. Using the patch-clamp technique developed by Erwin Neher and Bert Sakman (Neher and Sakmann 1976) (1991 Nobel Prize in Physiology or Medicine) it is possible to measure the current produced by the opening of a single ion channel molecule. The flux through an open Na^+^ channel of a nerve membrane, for example, is approximately 10^7^ ions s^−1^, which corresponds to a current of about 2 pA. Although this is a small current, it is nevertheless measurable. In contrast, ion pumps and transporters require significant conformational changes to move ions across a membrane against an electrochemical potential gradient. These conformational changes are necessary to avoid the ion binding sites being accessible to the solutions on both sides of the membrane simultaneously. As an analogy, one can think of an ion pump or transporter operating on the same principle as a lock on a river, where the gates of the lock are never opened to the river on both sides simultaneously. The conformational changes of pumps and transporters require many intermolecular forces between amino acid sidechains to be broken or formed, and they, therefore, occur on a much slower timescale than the opening of an ion channel. Typical turnovers of ion pumps or transporters are in the order of 100 s^−1^ or slower (Khalid and Clarke 2015). This corresponds to a current of about 0.02 fA, i.e., five orders of magnitude smaller than the current through a channel. To measure the currents produced by ion pumps or transporters, it is, therefore, advantageous to measure many of them simultaneously and synchronously. By attaching the membrane to a solid surface and measuring over a larger surface area, the observable currents of pumps and transporters would be much higher. This was a guiding principle in the development of a solid supported membrane (SSM) technique.

Fortunately for Klaus Fendler and *Herr* Bamberg, the group of Hermann Gaub at the Technical University of Munich had developed a method for SSM preparation (Florin and Gaub 1993), which Klaus and *Herr* Bamberg recognised should be compatible with the capacitive-coupling technique. The SSMs covered a surface area of approximately 0.25 cm^2^, i.e., 25 times greater than that of a BLM. Klaus and *Herr* Bamberg, together with PhD student Karsten Seifert, demonstrated proof-of-principle by measuring transient currents on Gaub’s SSM system using four different ion pumps: bacteriorhodopsin, the Na^+^,K^+^-ATPase, the H^+^,K^+^-ATPase and the sarcoplasmic reticulum Ca^2+^-ATPase (Seifert et al 1993). In each case the activation of the pumps was achieved through a laser flash, which activated bacteriorhodopsin directly and indirectly in the case of the ATPases by photolysing caged ATP.

Two years after these initial SSM measurements, at the beginning of 1995, I moved to Frankfurt to work in the Bamberg group. The previous five years 1990–94 I had been working in Berlin at the Fritz-Haber-Institute of the Max-Planck-Society in the group of Josef Holzwarth. In Berlin I was very fortunate to experience the fall of the Berlin Wall and German reunification first-hand, a major turning point in world history of the last century. Although *Herr* Läuger had died in 1990, he continued to have a decisive influence on my scientific career. Shortly before his death he wrote for me a reference letter, which, several years later, I was able to give to *Herr* Bamberg. No doubt this played an important role in *Herr* Bamberg’s decision to offer me a position at the Max-Planck-Institute in Frankfurt, where I had the best possible conditions to pursue my own research in parallel with and often in collaboration with Klaus. At that time Karsten Seifert was just leaving the institute, and I inherited his flat, in easy walking distance from the institute.

After the initial investigations using SSMs carried out during Karsten’s PhD, Klaus realised that the use of caged compounds placed too great a restriction on the method. In the same year as his initial SSM measurements he had carried out BLM measurements on the Na^+^,K^+^-ATPase where he corrected for the problem of incomplete photolysis of caged ATP and blockage of the enzyme’s ATP binding site by unphotolysed caged ATP (Fendler et al 1993), but no doubt he would have preferred to determine rate constant values directly from the raw data without the need to carry out a correction. He was also aware that caged compounds weren’t available for most transporter substrates. Therefore, he decided to combine SSMs with a rapid mixing technique, abandon the use of caged compounds, and thus develop a technique which has broader application to both ion pumps and secondary transporters. Klaus published the first measurements with a rapid solution exchange SSM technique based on capacitive coupling, i.e., the prototype of the SURFE^2^R, together with his PhD student Jürgen Pintschovius in 1999 (Pintschovius and Fendler, 1999). With this new technique they were able to carry out mixing experiments of the Na^+^,K^+^-ATPase with Na^+^ ions, which would have required a caged Na^+^ complex if they had continued along the photolysis route. The transient currents they observed on mixing with NaCl indicated that interaction of the Na^+^,K^+^-ATPase with Na^+^ on the cytoplasmic side of the membrane is an electrogenic reaction, i.e., that it involves charge displacement across the membrane (Pintschovius et al 1999). Using stopped-flow together with the voltage-sensitive fluorescent dye RH421 our group later showed that the same reaction also produced an RH421 fluorescent response, another indication of Na^+^-induced charge movement (Humphrey et al 2002). The result of Pintschovius et al 1999 was furthermore confirmed in Constance by Hans-Jürgen Apell and his PhD student Wolfgang Domaszewicz, who had also constructed a home-made SSM instrument of a different design (Domaszewicz and Apell, 1999) that allowed simultaneous measurement of capacitive coupling and the concordant response of the fluorescent dye RH421.

In 1998, at the same time as Jürgen Pintschovius was doing his PhD with Klaus in Frankfurt, a young Italian postdoc joined the Bamberg group, Francesco Tadini-Buoninsegni. Francesco came from the University of Florence, where he had completed his PhD in electrochemistry under the guidance of Rolando Guidelli and Maria Rosa Moncelli. While in Frankfurt, Francesco learnt how to use the new SSM device, and, on his return to Florence in 1999, set up his own home-made instrument in the laboratory. Until the commercialisation of the instrument (see next section), Florence and Frankfurt were the only locations in the world where SSM measurements were being performed on membrane transporters using an instrument of the Frankfurt design. In that time Francesco made significant contributions using the SSM to the transport mechanism of the sarcoplasmic reticulum Ca^2+^-ATPase, later to human and bacterial copper ATPases, and more recently to phospholipid flippases (Tadini-Buoninsegni et al 2008; Tadini-Buoninsegni 2020).

**Fig. 11:**
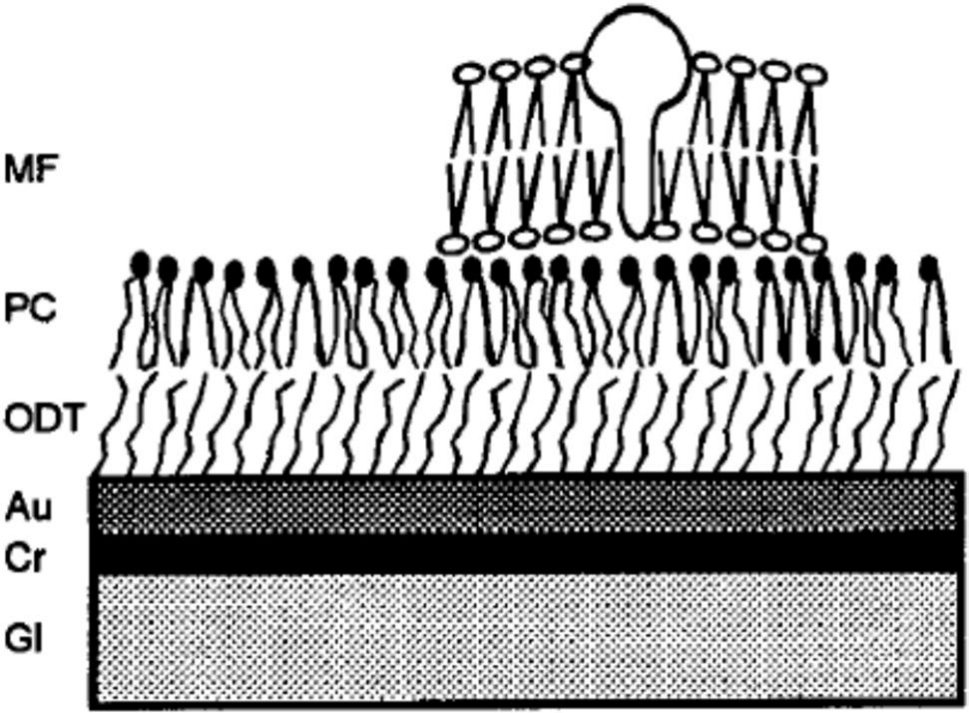
Structure of the solid supported membrane (SSM). The SSM consists of a glass support (Gl, 1 mm thick), a chromium layer (Cr, 5 nm thick), a gold layer (Au, 150 nm thick), an octadecanethiol monolayer (ODT), a diphytanoylphosphatidylcholine monolayer (PC), and the membrane fragments containing the protein (MF). Reprinted from the Biophysical Journal, 76, J. Pintschovius and K. Fendler, Charge translocation by the Na^+^/K^+^-ATPase investigated on solid supported membranes: Rapid solution exchange with a new technique, 814–826. Copyright 2025, with permission from Elsevier

The structure of the SSM of Klaus’s instrument is shown in Fig. 11. The stability of the SSM is predominantly due to the gold substrate to which the octadecanethiol forms a strong covalent bond via the sulphur of its thiol group. The alkane chains then form a basis for the self-assembly of a lipid monolayer above. The alkane and the lipid layers thus form two halves of a compound bilayer, onto which membrane fragments containing an ion pump or transporter can adsorb. The lipid used is normally diphytanoylphosphatidylcholine (DPhPC), which is a PC with methyl side groups along both of its hydrocarbon chains. This provides for strong van der Waals interactions between neighbouring lipid molecules, thus producing a relatively robust monolayer with no lipid phase transitions between 0 and 100 °C. The same lipid is also normally used for BLMs. To elicit a charge-transporting reaction of the membrane protein, an appropriate substrate of the protein is flowed across the SSM surface. The gold layer serves as one electrode and the second electrode is inserted into the solution bathing the SSM above the membrane fragments. Both electrodes are connected to the external measuring circuit, where current is measured via a current amplifier. Because the currents are so small, i.e., in the picoampere range, the entire system is enclosed in a Faraday cage to avoid external electrical interference. In the case of the original instruments, which Klaus had constructed in the institute’s workshops, the Faraday cage was a rectangular-shaped metal box, which colleagues thought resembled a child’s coffin *(Kindersarg* in German). Modern SURFE^2^R instruments have a more appealing appearance.

In Klaus Fendler, *Herr* Bamberg could not have had a better right-hand man. Klaus was an exceptional scientist, very practical, with an excellent knowledge of theory, open-minded and a fine mentor to the many students and postdocs in the Bamberg group. He was always very encouraging and supportive, with a good down-to-earth approach to science. I remember a saying of Klaus: “*Die Anderen kochen auch nur mit Wasser*” (i.e., the others also only cook with water), meaning that you’re as good as anyone else and can achieve just as good results. This is a great philosophy for any young scientist to have.

### Commercialisation

**Fig. 12:**
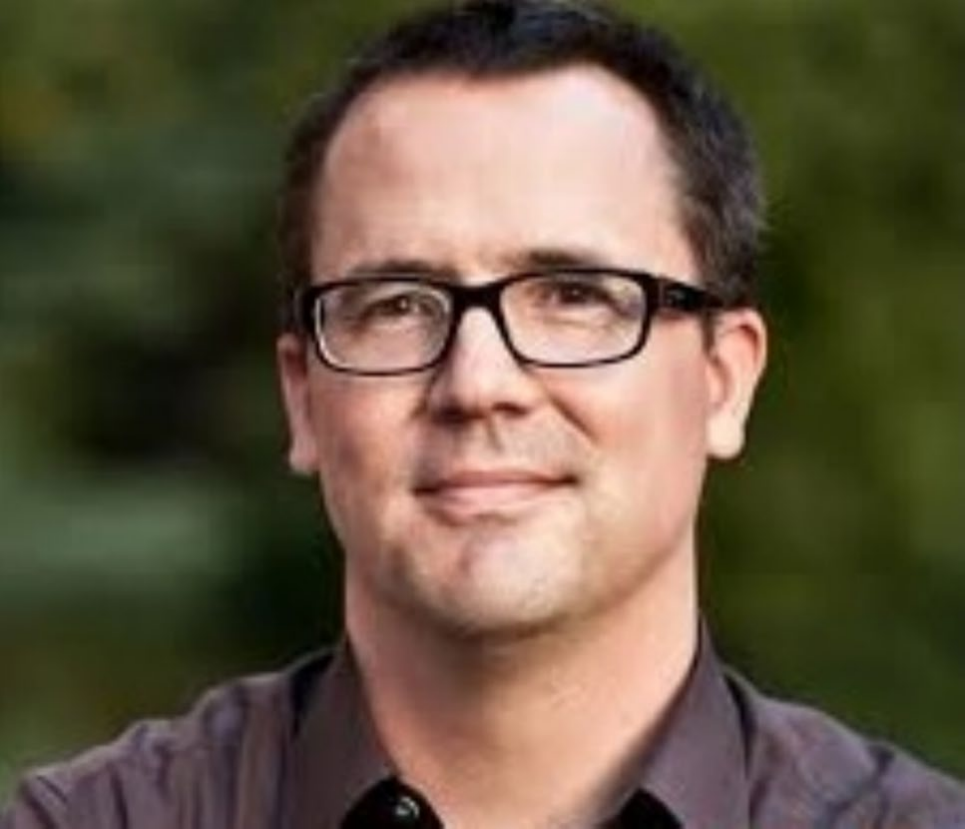
Thiemo Gropp, former PhD student in the Bamberg group of the Department of Biophysical Chemistry, Max-Planck-Institute of Biophysics. Thiemo founded the company IonGate Biosciences, which produced and commercialized the SURFE^2^R instrument. (photo courtesy of Thiemo Gropp)

If society is to gain any practical benefit from a scientific discovery or the development of a scientific instrument, sooner or later it needs to be commercialised. Only by commercialisation can sufficient funds be raised to allow an instrument to be more widely applied. In the case of the SURFE^2^R, the person who commercialised the instrument was Thiemo Gropp (see Fig. 12), a PhD student with Klaus at the Max-Planck-Institute of Biophysics. Thiemo had already completed his *Diplomarbeit* (equivalent of a B.Sc Honours thesis) at the institute in 1995, working together with Klaus Fendler in the Bamberg group. Because the institute is independent and not affiliated with a particular university, and only universities can confer degrees, Thiemo submitted his thesis to the Faculty of Physics at the University of Karlsruhe. The topic of his research was charge transport by the Na^+^,K^+^-ATPase, which he reconstituted into liposomes and then measured the protein’s kinetics on BLMs via capacitive coupling, releasing ATP from caged ATP by photolysis (Gropp et al 1998). After his *Diplomarbeit,* Thiemo stayed at the institute for his PhD but switched his protein of interest from the Na^+^,K^+^-ATPase to the ADP/ATP carrier of the mitochondrial membrane (Gropp et al 1999). This was a collaboration with Martin Klingenberg at the Ludwig-Maximilians-University of Munich.

During his PhD Thiemo must have realised the potential of the SSM technique. After he finished his degree, he founded the company IonGate Biosciences. With the commercial funds that he then had available to him, he developed the SSM technique into the modern SURFE^2^R instrument (**Surf**ace **E**lectrogenic **E**vent **R**eader). The new company rented office and laboratory space in the west of Frankfurt on the grounds of the chemical company Hoechst (later, after a merger, Aventis, and then after another merger Sanofi-Aventis). The Bamberg group at the Max-Planck-Institute, of which Thiemo had been a member, was always a tightly knit group of people. The PhD students and postdocs would often socialise together on weekends and in the evenings. At that time (late 1990s), the Biophysical Chemistry department of the institute was located in the former Tudor-style villa of a rich Jewish banker in the Frankfurt suburb of Sachsenhausen. In 1938 the villa was provided to the Max-Planck-Society to house the newly formed Max-Planck-Institute for Biophysics and was converted for scientific use (Gerwin 1982). It was a beautiful building in which to do research, but also to socialise. There was a kitchen and dining hall where the group often cooked and ate together. *Herr* Bamberg contributed to the good social atmosphere within the group. Every two years he would organise a group seminar to the “Max-Planck-Castle”, *Schloss Ringberg*, on the Tegernsee in Upper Bavaria. Foreign collaborators were also often invited to attend these events, e.g. Flemming Cornelius (Denmark), Jeff Froehlich (USA), Constanta Ganea (Romania), and Hemi Gutman (Israel). Because of Thiemo’s experience in the Bamberg group, it’s not surprising that when he founded IonGate, he recruited a number of other former PhD students from the group to come and work with him: Bela Kelety, Natalie Watzke, Maarten Ruitenberg and Andreas Haase. Not only were they already experienced with the SSM technique, they also had an excellent already-established working and personal relationship.

As described in the previous sections, the focus of biophysical research using the capacitive-coupling technique and SSMs had been to gain fundamental knowledge about the mechanisms of ion pumps, such as bacteriorhodopsin and the Na^+^,K^+^-ATPase. With Thiemo at the helm, IonGate shifted this focus more strongly towards the establishment of assays for pharmacologically relevant proteins, such as bacterial transporters, and the screening of drug molecules for transporter inhibition. Of course, this expansion of the application of the SURFE^2^R was important for IonGate from a financial point of view, but it also shifted the benefits of the technique from solving questions of purely scientific interest into the medical field of drug development for the treatment of diseases, from which society in general can gain more benefit.

After the founding of IonGate in 2000, the first few years of the company were devoted to technical development and improvement of Klaus’s SSM electrophysiological technique. This included robotizing the flow system for solution uptake and injection across the SSM surface and improving the instrument’s computer control. These improvements significantly reduced the time required to perform experiments. In parallel, researchers within the company were investigating other applications of the instrument, in particular in the area of assays for drug development (Kelety et al 2006; Geibel et al 2006). Such assays were greatly facilitated by the robotization of the instrument. In addition, IonGate developed a multi-well-plate version of the SSM instrument, which is even more suited to drug discovery applications. In 2006, the first model of the SURFE^2^R instrument was launched by IonGate. Drug development is now the main application of the instrument, both in industry and academic research. According to review papers, drug assays for over 100 different membrane proteins have now been established using the SURFE^2^R (Tadini-Buoninsegni and Fendler 2015; Bazzone et al 2017; Tadini-Buoninsegni and Palchetti 2020; Bazzone and Barthmes 2020; Pommereau et al 2023).

### Nanion

**Fig. 13:**
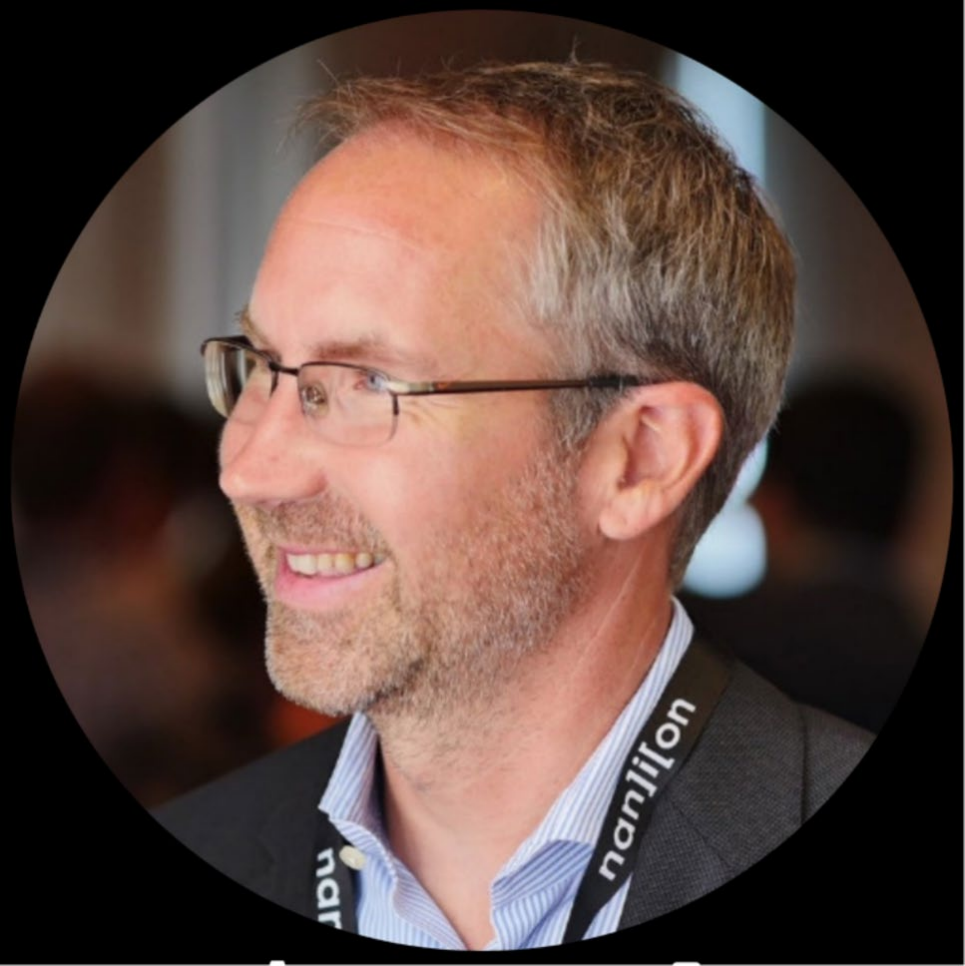
Niels Fertig, CEO of Nanion Technologies. Niels founded the company in 2002 as a spin-off from the Ludwig-Maximilians-University Munich, Germany. Nanion today manufactures and provides the SURFE^2^R instrument (photo courtesy of Niels Fertig)

The company Nanion Technologies was founded in 2002 by Niels Fertig (see Fig. 13) as a spin-off of the Center for Nanoscience of the Ludwig-Maximilians-University of Munich (LMU). At that time Niels was still doing his PhD in Biophysics at the Center. His supervisors were officially Joerg Kotthaus and Hermann Gaub, but he worked most closely with Jan Behrends, a postdoc in the Institute of Physiology at the LMU at the time (later Professor of Physiology in Freiburg). In his PhD research Niels was focussed on electrophysiological measurement of ion channels via single-channel and whole-cell versions of the patch-clamp technique, originally developed by Neher and Sakmann (Neher and Sakmann 1976). One of the issues which limited the wider application of this technique was the skill required to manipulate the patch pipette under the microscope to attach the pipette to a single cell. Niels designed an approach whereby the patch pipette is replaced by a perforated planar glass substrate to which the cell adheres (Fertig et al 2002a,b). This significantly reduced the difficulty of the technique and stimulated its wider application. This success was the springboard for the foundation of Nanion. In 2003 the company launched its Port-a-Patch system, a miniaturised planar patch-clamp system based on the systems Niels had designed during his PhD.

**Fig. 14:**
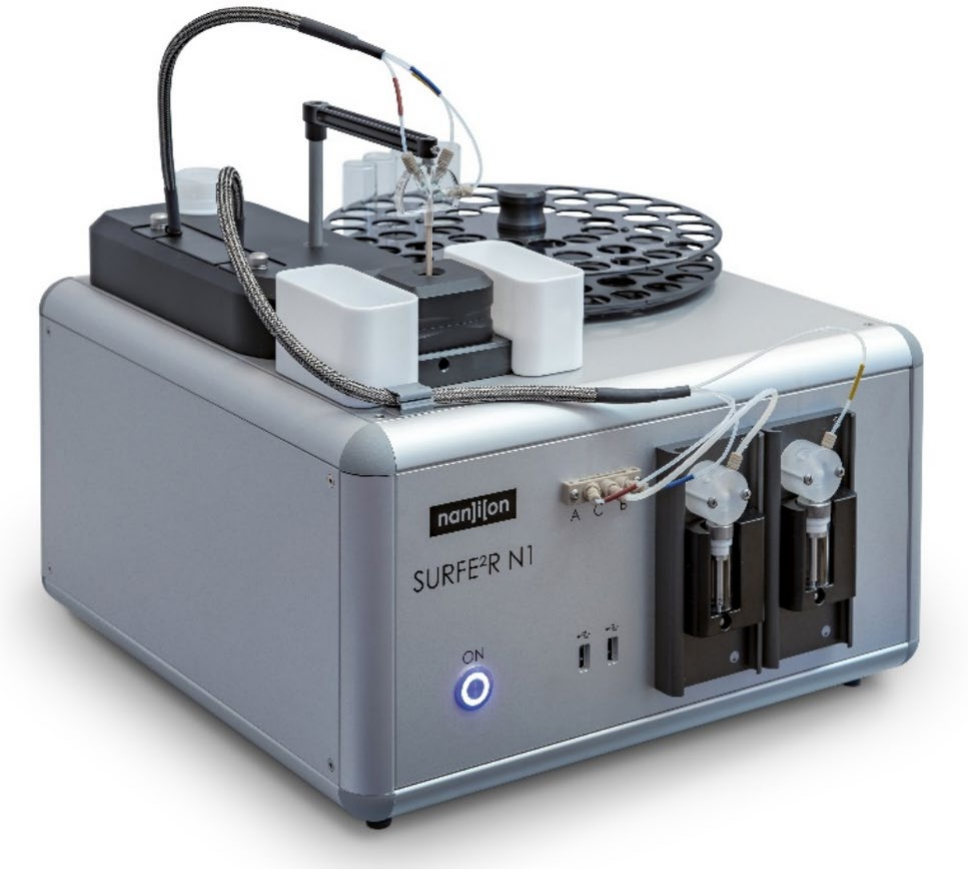
The SURFE^2^R, model N1, of Nanion Technologies. Image provided by Dr Maria Barthmes, Nanion Technologies

In the world today there are many more researchers working on ion channels than on ion pumps or transporters. This does not necessarily mean that ion channels are more important than pumps and transporters, but, as described earlier, ion channels produce much larger currents across membranes than pumps and transporters, and hence ion channels are much easier to investigate experimentally. Because of this, the market for a new instrument to investigate ion channels, such as the Port-a-Patch, is much greater than the market for a new instrument to investigate pumps and transporters, such as the SURFE^2^R. IonGate was founded only two years before Nanion, but because of the difference in the breadth of their market, Nanion had the better financial outlook. With its foundation in ion channels, Nanion was then later able to expand its market into pumps and transporters by buying out the rights to the SURFE^2^R when IonGate ceased operation due to financial difficulties. In fact, it was Thiemo Gropp from IonGate who first approached Niels to ask if Nanion would be interested in taking over the handling of the SURFE^2^R instrument. The two of them met at Nanion’s offices in Munich and they quickly came to a good agreement. Thus, Nanion effectively secured a future for the SURFE^2^R when it was in danger of extinction. They relaunched the instrument on the market in 2012. Since then, Nanion has made further improvements, including a redesigned internal structure, an improved measurement chamber, and optional light stimulation for light-activated transporters in addition to the rapid flow system. A photo of the SURFE^2^R instrument, model N1, as it appears today is shown in Fig. 14.

After acquiring the SURFE^2^R from IonGate in 2012, Nanion needed someone as a product specialist for the instrument. At the time Maria Barthmes was working on her PhD in Pharmacology at the LMU. The focus of her thesis was the development of new electrophysiological methods for the investigation of prokaryotic and mitochondrial systems. This was a collaboration between the LMU and Nanion, so naturally Maria spontaneously started working with the SURFE^2^R as soon as it came to Nanion. In addition, with a background in bioengineering from her Master’s degree, Maria was the ideal person to become Nanion’s new SURFE^2^R product specialist. Niels appointed her to this role in 2012, even before she’d completed her PhD. In 2015, after her graduation, she then became product manager. One year later, in 2016, André Bazzone joined Nanion’s SURFE^2^R team. André had just completed his PhD with Klaus Fendler at the Max-Planck-Institute of Biophysics in Frankfurt, where he’d gained extensive experience in SSM measurements. Similar to Maria, for André the timing could not have been better. Nanion wanted to build up its SURFE^2^R team just at the time that he completed his PhD. On the recommendation of Klaus, André was able to walk straight into a job with Nanion.

**Fig. 15:**
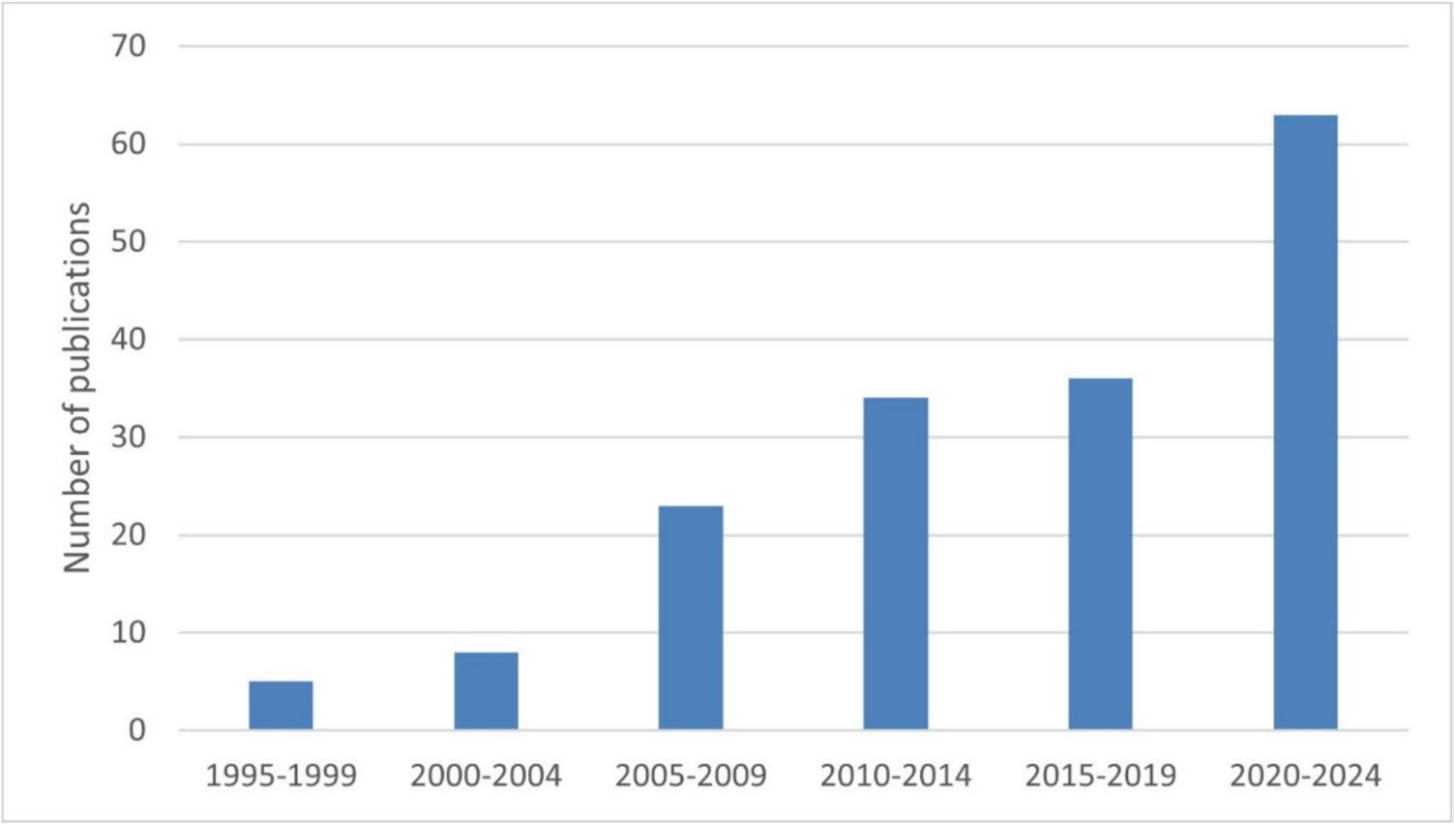
Increase in scientific publications using capacitively-coupled SSM technology since its invention in the 1990s

Since its invention in Klaus’s laboratory in the 1990s, use of the capacitively-coupled SSM technique and its subsequent commercial version, the SURFE^2^R, has been increasing exponentially (see Fig. 15). As mentioned earlier, a major reason for the increasing use of the technique is its application to drug screening. As an example of a medical application of the SURFE^2^R, an assay was recently reported (Pommereau et al 2024) where the SURFE^2^R could be used as a method of high-throughput screening of drugs for their efficacy in inhibiting the membrane transporter GLUT9, which is responsible for reabsorption of uric acid into the bloodstream in the kidney. Elevated blood serum levels of uric acid are associated with gout, hypertension, insulin resistance, and cardiovascular and kidney diseases. In the 1970s it could not have been foreseen that research into finding a better way to measure the proton pumping activity of bacteriorhodopsin would eventually lead to an instrument now being used to discover drugs to treat cardiovascular disease. This stresses how important it is that governments fund fundamental research where the later application may not be immediately obvious. I’m sure that scientists reading this article can think of other examples where fundamental research has later led to practical applications, but it’s a lesson that politicians sometimes forget or prefer to ignore (Clarke 2023a, b).

### Fundamentals of SURFE^2^R operation

**Fig. 16:**
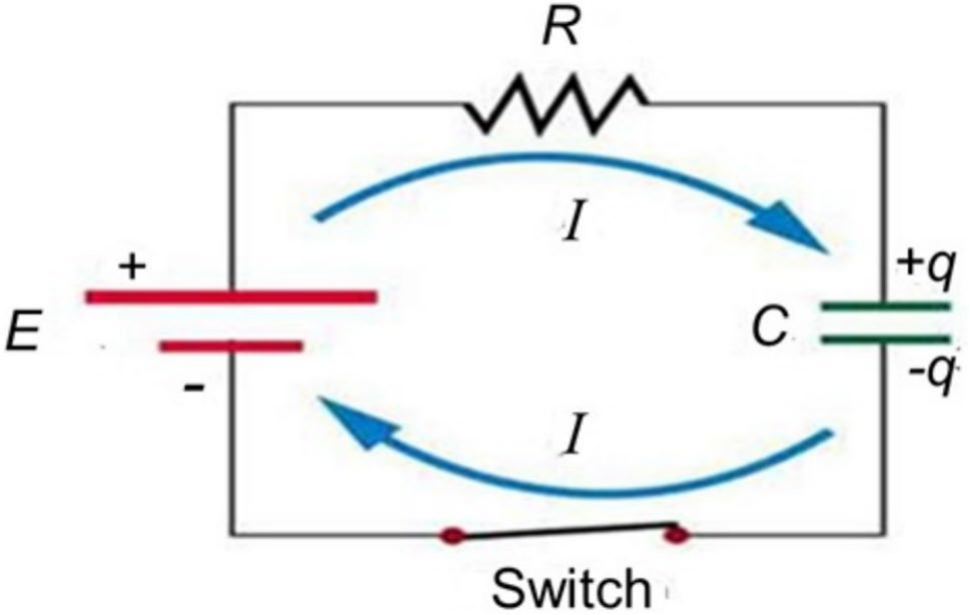
An electrical circuit with a power supply, *E*, and a resistor, *R*, and capacitor, *C*, in series. *I* is the current (i.e., direction of flow of positive charge) and *q* is the charge built up on the plates of the capacitor after closing the switch

**Fig. 17:**
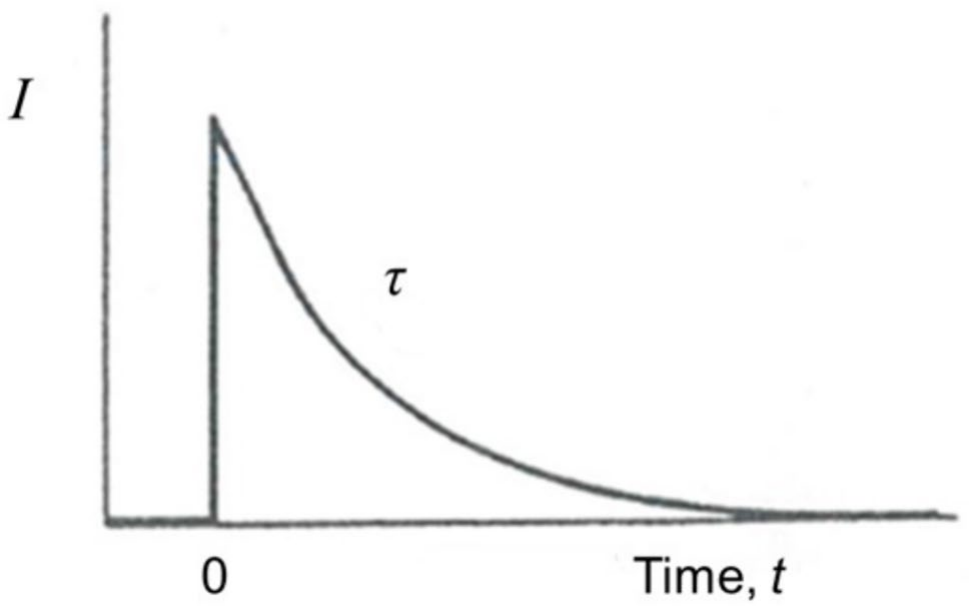
Time course of the current in the electrical circuit shown in Fig. 15 after the switch has been closed at time = zero. A transient current occurs until the capacitor is fully charged. The current decays exponentially from the current at time zero, *I*_0_. At the relaxation time or time constant of the circuit, τ, the current has decayed to *I*_0_/e, i.e., *I*_0_/2.72

As described earlier, the SURFE^2^R instrument is based on the principle of capacitive-coupling. Before discussing measurements on membrane transporters or pumps, the easiest way to explain the method is to start with the simple electrical circuit shown in Fig. 16. When the switch is closed, current starts to flow in the circuit. No charge can jump between the plates of the capacitor because the dielectric medium between them is assumed to be nonconducting. Nevertheless, current flows because positive charge builds up on the upper plate, which creates an electric field, forcing positive charge off the bottom plate. Thus, the two plates gain equal and opposite charges. Immediately after closing the switch the current will be a maximum, but as the charges build up on the two plates of the capacitor, the electric fields created by the charges will push positive charge back away from the top plate and draw positive charge back onto the bottom plate of the capacitor. Therefore, the current will be at a maximum immediately after closing the switch and it will gradually decay back to zero as the capacitor becomes fully charged (see Fig. 17). Note that current in Fig. 16 is defined classically as the rate of flow of positive charge, whereas in actual fact the charge carriers are the negatively charged electrons.

The decay of the current, *I*_*t*_, in the circuit follows an exponential time function, as described by Eq. 1, with a time constant, τ, given by the product of the resistance and capacitance, *RC*, of the circuit.

1 $${I}_{t}={I}_{0}\,exp\left(-\frac{t}{\tau }\right)$$

*I*_0_ is here the initial current immediately after closing the switch.

**Fig. 18:**
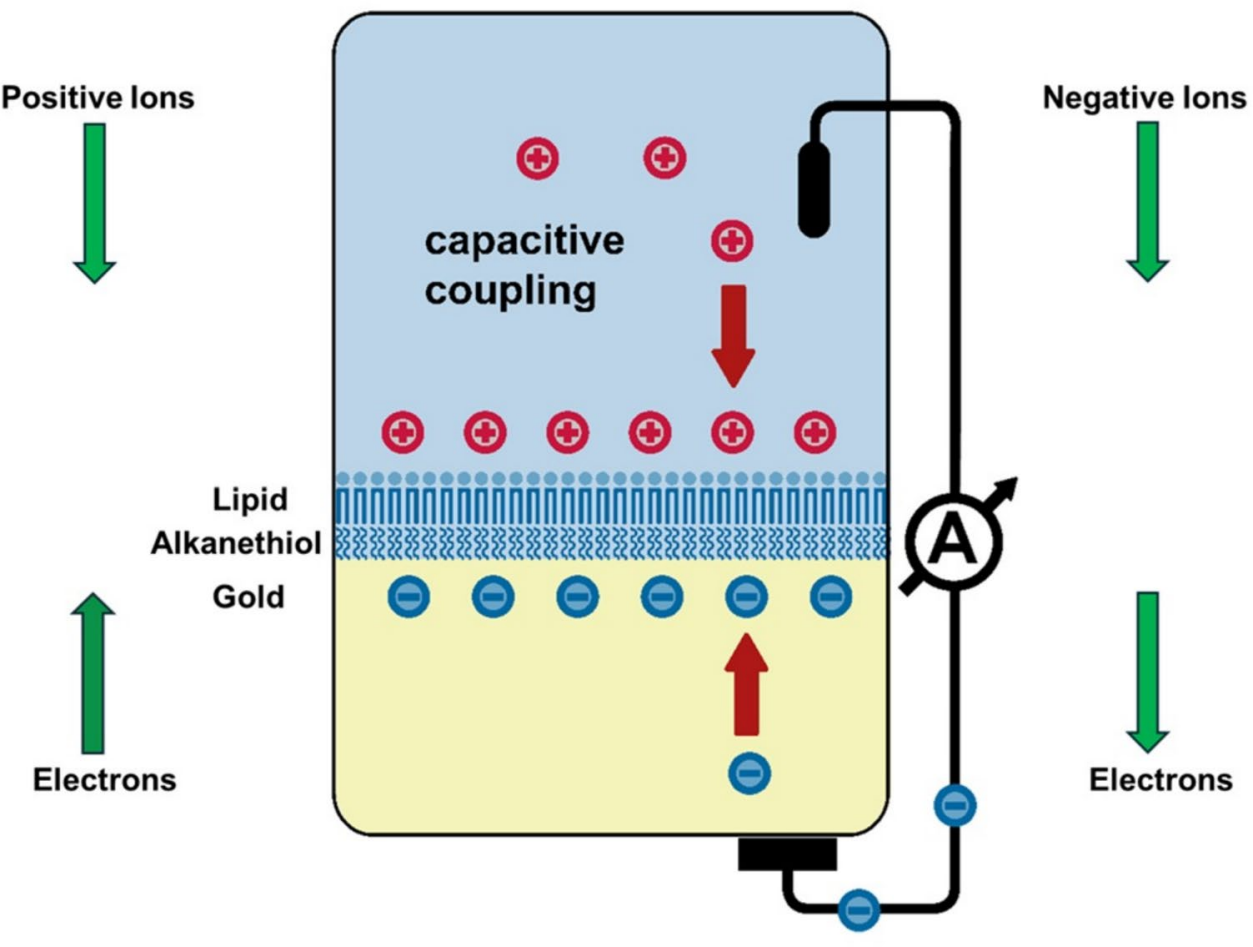
The electrical circuit of the SURFE^2^R instrument. The centre of the figure shows positive ions (cations) binding to the membrane. To compensate for the build-up of positive charge on the membrane (i.e., on one side of the capacitor) electrons flow through the external measuring circuit towards the gold substrate (i.e., the other side of the capacitor). If negative ions (anions) bind to the membrane the situation is reversed. Then electrons flow away from the gold substrate to produce a compensating positive charge on the gold substrate

Now let us consider the circuit of the SURFE^2^R instrument and compare it with the simple circuit shown in Fig. 16. For simplicity we’ll initially consider the case of the bare compound membrane of the SURFE^2^R with no membrane fragments or vesicles containing proteins adsorbed. The relevant circuit is shown in Fig. 18. Here we are considering the interaction of the SURFE^2^R membrane with ions that can bind to the membrane surface, e.g. due to an inherent lipophilicity of the ions or to a charge which allows them to interact electrostatically with the lipid head groups. It is possible that a small background stationary current due to the resistance component of the membrane in parallel with its capacitance (see Fig. 4), but for simplicity here we ignore this component of the current signal.

The alkanethiol/lipid compound membrane of the SURFE^2^R is analogous to the capacitor in the simple electrical circuit shown in Fig. 16. However, the SURFE^2^R circuit has no power supply. The power supply is substituted by a pump which forces charged ions (rather than electrons in the electrical circuit of Fig. 16) through the flow circuit towards the membrane surface. Of course there is no net current through the flow circuit, because the number of cations and anions in the solution are equal, i.e., the solution is electrically neutral. However, if ions of a certain charge bind preferentially to the membrane, this is equivalent to charge building up on one plate of a capacitor. Just as in the case of a parallel plate capacitor, the charge on one plate must be compensated by an equal and opposite charge on the other plate. Therefore, if positive ions bind preferentially to the membrane, electrons must flow through the measuring circuit to the gold surface. If negative ions bind preferentially to the membrane, electrons must flow away from the gold surface. Either way, an electrical current is induced in the external part of the circuit which reflects what is happening at the membrane surface.

**Fig. 19:**
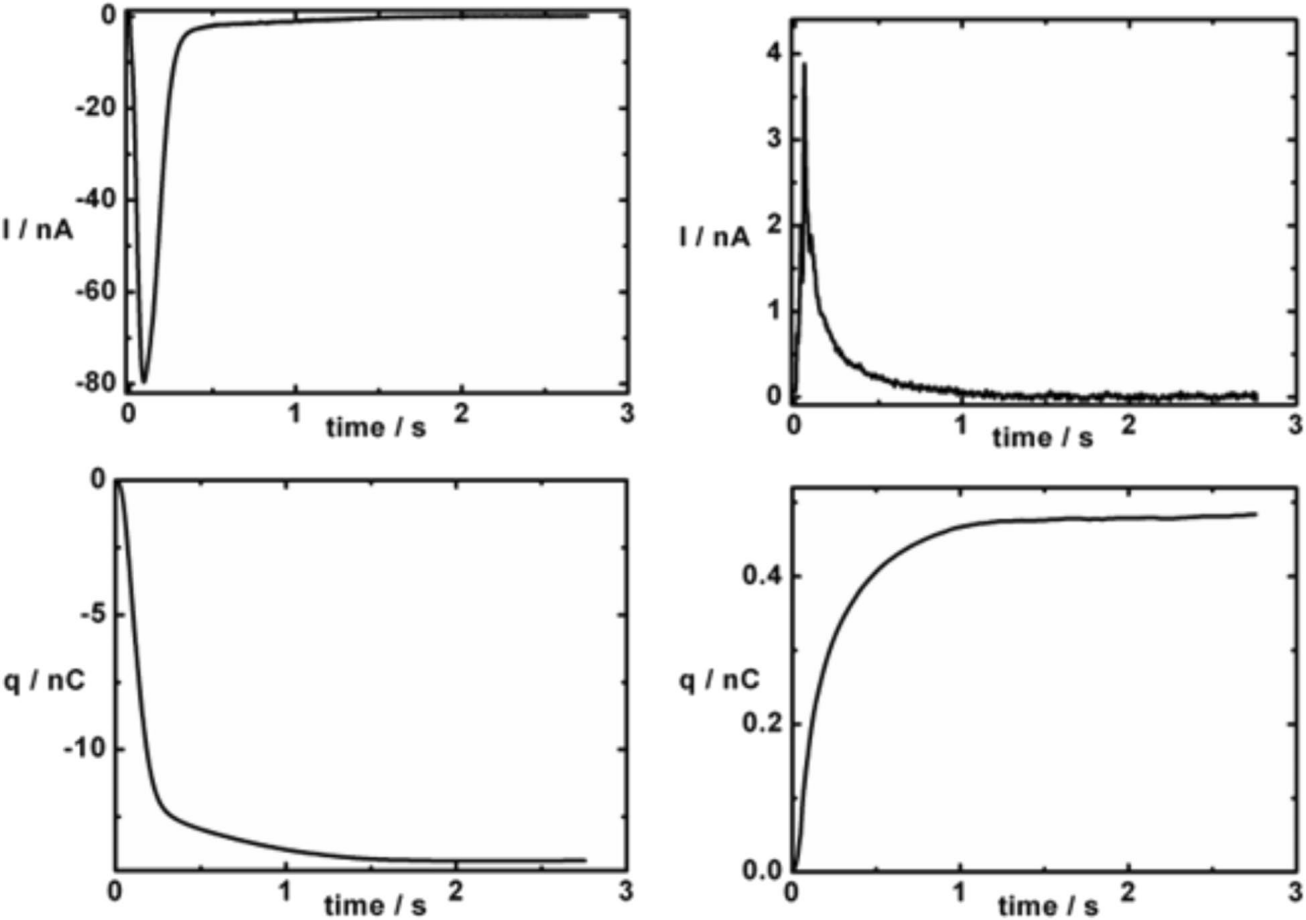
Upper two panels: transient currents, *I*, produced in the SURFE^2^R due to binding of the hydrophobic ions, tetraphenylborate (TPB^─^, left) and tetraphenylarsonium (TPA^+^, right) to the SURFE^2^R’s compound membrane. Because of the opposite charges of the two ions, the polarities of the currents are opposite. TPB^─^ is negatively charged and produces a negative current. TPA^+^ is positively charged and produces a positive current. The concentrations are 100 μM for TPB^─^ and 200 μM for TPA^+^. Lower two panels: the charge, *q*, adsorbed to the membrane as a function of time for TPB ─ (right) and TPA+ (left). In the case of both ions, the amount of charge adsorbed gradually increases until the reaction of the ion in the bathing solution reaches an equilibrium with the membrane. The total amount of charge bound at equilibrium corresponds to approximately −14 nC for TPB ─ and + 0.5 nC for TPA+. TPB ─ binds more strongly to phosphatidylcholine membranes than TPA+, which accounts for the greater charge adsorption by TPB ─. The curves of q versus t have been obtained by integrating the upper curves as a function of time. It can clearly be seen that the TPB ─ curve is biphasic, i.e., not a single exponential. This indicates that the reaction of TPB ─ with the membrane occurs in at least two steps. These are attributed to TPB ─ binding rapidly to the membrane surface followed by a slower diffusion across the membrane. Reprinted with permission from Baumgart et al. (2025) Membrane binding of hydrophobic ions: Application of new kinetic methods. Langmuir 41:8081–8091. Copyright 2025 American Chemical Society

Examples of measurements are shown in Fig. 19. Because of their surrounding phenyl rings, the ions tetraphenylarsonium and tetraphenylborate are hydrophobic and bind well to lipid membranes, inducing relatively large currents that one can easily measure (Baumgart et al 2025). If one adsorbs membrane sheets or vesicles containing an ion-transporting membrane protein, e.g. an ion pump or a transporter, on top of the compound membrane and a charge movement is caused relative to the surface of the membrane sheets or vesicles by the ion-transporting activity of the pump or transporter, this also induces a current in the external measuring circuit. Researchers interested in membrane proteins often regard ion binding to the SURFE^2^R’s compound membrane as a nuisance because it produces a current which overlays that produced by the protein. This can make it difficult to isolate the signal due to the protein alone. However, studies on ion binding to membranes are also very useful. In his group Klaus Fendler studied the interaction of many cations and anions with solid-supported membranes (Garcia-Celma et al 2007). The sequence of ion affinities to the membrane agreed well with previous studies using lipid vesicles and detecting ion binding via the voltage-sensitive dye RH421 (Clarke and Lüpfert 1999). Therefore, it seems clear that the SURFE^2^R has broader application than just to ion transport proteins. It could also be a valuable tool for researchers in colloid and surface chemistry more broadly. Furthermore, it has potential application in drug discovery. Firstly, it can be used to test the effectiveness of drugs in inhibiting the activity of ion pumps and transporters, particularly those of microbial origin. Secondly, drugs able to stimulate transport activity of ion pumps and transporters are also an interesting research topic, since pharmacological stimulation of transporter activity represents a promising therapeutic strategy in various disease states. Thirdly, the SURFE^2^R could be used as a screening method for drug interaction with cell membranes. Currently the most commonly used method to estimate the lipophilicity of a drug and its ability to cross a cell membrane is to measure its octanol/water partition coefficient. But the headgroup of a phospholipid is very different from that of octanol, which is just a hydroxyl group. Therefore, binding studies using the SURFE^2^R could be a more reliable method of determining membrane interaction of charged drug molecules.

A final important point regarding the fundamentals of SURFE^2^R operation is how one should analyse the transient current data obtained. For experiments in which one wishes to determine the dissociation constant of an ion to the membrane or to a membrane protein from a titration of the ion, one method is to plot the peak current as a function of the ion concentration and then fit to a hyperbolic binding curve. Because current is the rate of flow of charge, i.e. d*q*/d*t*, the integral of the entire transient current curve gives the total charge bound. Instead of using the peak current, the integral of the curve, i.e. *Q*_tot,,_ can be plotted as a function of concentration and then, as in the case of the peak current, fit the data to a hyperbolic binding curve.

In principle, it is also possible to obtain kinetic data from SURFE^2^R measurements. Hussein et al (2024) developed a numerical model to simulate the transient current data generated by the SURFE^2^R instrument and used the model to reproduce data obtained from the ATP-dependent K^+^ pump KdpFABC. A different approach was recently followed by Baumgart et al (2025). As just explained, the raw data obtained in any capacitive-coupling technique is a transient current, which is already a rate. This is different from many other kinetic techniques, where one measures concentration as a function of time, or some observable which is proportional to concentration, such as absorbance or fluorescence. An example here would be the stopped-flow technique, which, assuming one is using optical detection, yields kinetic curves of absorbance or fluorescence as a function of time. Then one fits the measured traces to one or more exponential functions to determine observed rate constants or reciprocal relaxation times. For the analysis of transient currents via the same approach, one first needs to integrate the transient current curves with respect to time to obtain *q*(*t*), i.e., the total amount of bound charge at every point in time:

2 $$q\left(t\right)={\int }_{0}^{t}I\left(t\right)$$

Once this has been done, the *q*(*t*) versus *t* curve can be fitted to a sum of exponential functions in the same way as in the analysis of stopped-flow curves. This procedure has been used to analyse the kinetics of TPB^─^ and TPA^+^ interaction with the SURFE^2^R membrane (Baumgart et al, 2025) (see Fig. 19) and show that the interaction occurs via a two-step process, i.e., rapid binding to the membrane surface followed by slower diffusion across the membrane.

## Conclusion

It has been a pleasure to have the opportunity to write this paper and honour the hard work of the relatively small number of friends and colleagues who have contributed to bringing the SURFE^2^R from a concept of fundamental scientific research to a commercial instrument that is now available for use by researchers in different fields the world over. I am sure that this story of the SURFE^2^R is not unique. Any experimental technique that is to make it into commercial production needs its inventors and its champions, but often we don’t hear about the hard work that has gone on behind the scenes before an instrument is marketed. I hope that the reader has enjoyed reading about the scientific development of the SURFE^2^R as well as the personal side of the research involved. I hope this story will encourage others to consider whether the SURFE^2^R has an application in their own research and to appreciate the effort that has gone into creating other instruments that they might have in their laboratory. Finally, I hope that the story of the SURFE^2^R, with its origins in fundamental scientific research, leading many years later to pharmacological research within pharmaceutical companies, searching for drugs to treat human disease, serves as an example for funding agencies of the importance of financially supporting fundamental research.

## Data Availability

No datasets were generated or analysed during the current study.
